# My social comfort zone: Attachment anxiety shapes peripersonal and interpersonal space

**DOI:** 10.1016/j.isci.2023.105955

**Published:** 2023-01-11

**Authors:** Mariana von Mohr, Paulo C. Silva, Eleonora Vagnoni, Angelika Bracher, Tommaso Bertoni, Andrea Serino, Michael J. Banissy, Paul M. Jenkinson, Aikaterini Fotopoulou

**Affiliations:** 1 Department of Psychology, Royal Holloway, University of London, London, UK; 2 School of Life and Medical Sciences, University of Hertfordshire, London, UK; 3 Research Department of Clinical, Educational and Health Psychology, University College London, London, UK; 4 Department of Psychology, Bournemouth University, Bournemouth, UK; 5 Max Planck Institute of Human Cognitive and Brain Sciences (IMPRS), Leipzig, Germany; 6 Department of Child and Adolescent Psychiatry, Psychotherapy and Psychosomatics, University of Leipzig, Leipzig, Germany; 7 MySpace Lab, Department of Clinical Neuroscience, Lausanne University Hospital (CHUV), University of Lausanne, Laussane, Switzerland; 8 School of Psychological Science, University of Bristol, Bristol, UK; 9 ISN Psychology, Institute for Social Neuroscience, Melbourne, VIC, Australia

**Keywords:** Social medicine, Behavioral neuroscience

## Abstract

Following positive social exchanges, the neural representation of interactive space around the body (peripersonal space; PPS) expands, whereas we also feel consciously more comfortable being closer to others (interpersonal distance; ID). However, it is unclear how relational traits, such as attachment styles, interact with the social malleability of our PPS and ID. A first, exploratory study (N=48) using a visuo-tactile, augmented reality task, found that PPS depended on the combined effects of social context and attachment anxiety. A follow-up preregistered study (N = 68), showed that those with high attachment anxiety demonstrated a sharper differentiation between peripersonal and extrapersonal space, even in a non-social context. A final, preregistered large-scale survey (N = 19,417) found that people scoring high in attachment anxiety prefer closer ID and differentiate their ID less based on feelings of social closeness. We conclude that attachment anxiety reduces the social malleability of both peripersonal and interpersonal space.

## Highlights

• No evidence that affective touch modulates peripersonal space
• Attachment anxiety sharpens peripersonal space, irrespective of social context
• Attachment anxiety increases comfort with social proximity, irrespective of intimacy
• Attachment anxiety reduces the social malleability of peri- and interpersonal space

## Graphical abstract

**Figure:**
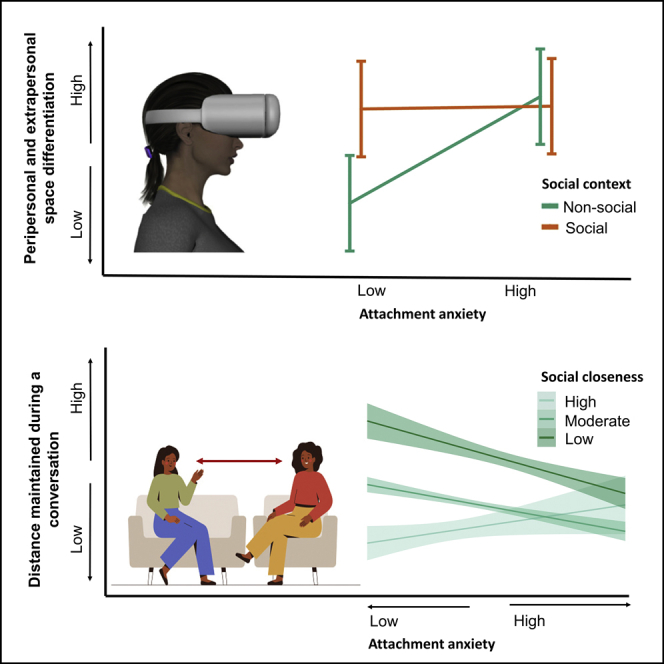

## Introduction

Seminal, sociological studies have suggested that people maintain a kind of safety zone around the body during social interactions, a so-called personal, or interpersonal space, that depends on the type, strength, and cultural meaning of the social relationships and interactions at stake.^1^^,^^2^^,^^3^^,^^4^ New lines of research have tested these ideas using novel social manipulations of the distance maintained between individuals in everyday life (termed preferred comfort distance^5^^,^^6^^,^^7^), as well as social manipulations during an audio-tactile interaction paradigm that measures the extent of PPS,^8^ i.e., the multisensory representation of space surrounding the body encoded by a set of neurons in frontoparietal areas of the brain^9^ responsible for generating defensive responses to threats in the local environment,^10^^,^^11^ and for responding to stimuli that are near or approaching the body.^12^ Although it remains debated whether (inter)personal space, as indexed for example by preferred comfort distance, and PPS, as measured by multisensory integration paradigms, are processed by similar or different psychological and neural mechanisms (reviewed by^13^), recent research has increasingly highlighted that social-affiliated factors are crucial for the instantiation not only of our interpersonal space but also our PPS. For example, although our PPS shrinks by the presence of a stranger,^14^ it expands after we perceive interpersonal exchanges to be prosocial and trustworthy,^12^ and after a collaborative positive social interaction^14^^,^^15^ (but see^16^), to create a space for interaction (see^17^ for a review). However, little is known about how this malleability of our PPS based on social context, as well as the more sociological notions of interpersonal space, such as the distance people are comfortable maintaining during conversations,^1^^,^^2^^,^^18^^,^^19^ can be shaped by key individual traits of interpersonal relating. Specifically, although it is known that PPS can be influenced by trait anxiety,^20^^,^^21^ and incursions in one’s personal space are accompanied by discomfort and anxiety^22^ (see also^23^ but note a different PPS paradigm was used*)*, it is unknown how the social modulation of PPS, as well as related everyday notions of interpersonal space, can be influenced by attachment anxiety. Distinct from clinical anxiety, attachment anxiety is a key dimension of attachment style, i.e., internal working models of social relating and associated hyperactivating, affect regulation strategies, which are thought to be developed since early childhood in response to differences in the responsiveness and availability of caregivers to their infants’ needs.^24^ Of interest, a recent study suggests that the parieto-frontal cortical system that monitors PPS is heightened in anxiously attached individuals.^25^ More generally, the role of such individual differences may be important because attachment anxiety is known to introduce negative biases in the interpretation of social cues and to influence particularly how trustworthy social partners are perceived to be.^26^ Specifically, higher levels of attachment anxiety can lead to heightened worry about emotional closeness and abandonment, with related hypervigilance, mistrust and persistent seeking and checking for signals (evidence) of support or ‘non-rejection’ from others^27^ and a hence distinctive lack of comfort, or ease during interpersonal interactions. Even though PPS and interpersonal distance are different constructs, they are related^28^^,^^29^ with the former modulating the latter^30^ and critically, the little research available on personal space and attachment anxiety suggests a relationship between attachment style and interpersonal distance. For example, the higher the attachment anxiety the more the chosen interpersonal distance, as measured by the stop-distance paradigm to assess tolerance for interpersonal proximity.^31^ This latter finding was not predicted by the original authors given that proximity seeking is typically associated with attachment anxiety, but was interpreted by the original authors as a potential consequence of the relational mistrust also associated with attachment anxiety and a finding that warrants further research. Accordingly, here we expected that the higher the attachment anxiety, the less defined the PPS and the closer the preferred interpersonal distance but particularly in social settings characterized by active support or perceived close intimacy given that only such conditions can provide some evidence of the desired certainty about social proximity, and vice versa.

**Figure 1:**
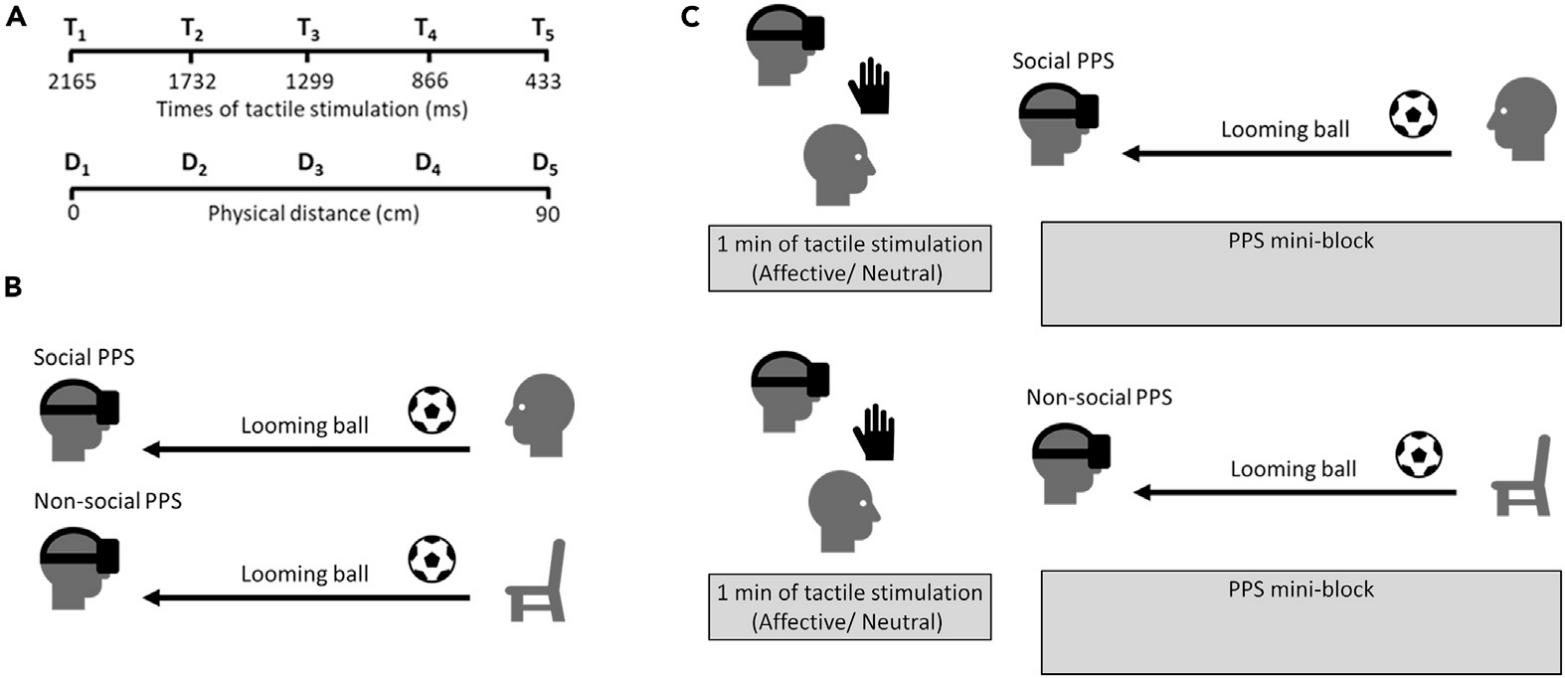
Peripersonal space (PPS) task (A) Visual representation of the relation between time and space in the PPS task. In the trials where the looming ball is present, it will appear at the beginning of the trial and gradually approach the participant. The tactile stimulation will happen at specific time delays from the trial onset (bimodal trials). These times will therefore correspond to virtual distances between the ball and the participant. The bigger the delay, the closer the ball will feel to the participant (Time 1 to 5 and Distance 1 to 5, respectively). In some other trials, participants received the tactile stimuli at the same time intervals but no ball was presented (tactile unimodal) or the ball was presented without any tactile stimuli respectively (visual unimodal). See STAR Methods section for details. (B) Social and non-social PPS conditions. In the social PPS condition there was the presence of another person (experimenter) sitting in front of the participant and in the non-social there was no one. This manipulation was done between-subjects for Study 1 and within-subjects for Study 2. (C) Example of one mini-block in Study 1. There were 10 mini-blocks in each block, where participants received slow, affective (at 3 cm/s) or very slow, neutral (at 0.3 cm/s) touch depending on the block (within-subjects manipulation). Participants were instructed to close their eyes during the slow and very slow tactile stimulation. Tactile stimulation was followed by 18 PPS trials, which included three types of trials, namely bimodal visuo-tactile, unimodal tactile and catch trials. Half of the participants were assigned to the social PPS group (upper level) and the other half to the non-social PPS group (lower level).

To address this question, i.e., what is the role of individual differences in attachment anxiety in the social modulation of PPS and interpersonal space, we conducted a series of three studies; an initial exploratory study followed by two well-powered, preregistered studies. We tested how attachment anxiety modulates PPS (Studies 1 and 2) and interpersonal space (Study 3) change as a function of embodied social support (i.e., the receipt of affective versus non-affective touch, because this mode of support has shown optimal results in other domains,^32^: Study 1) and social context (i.e., presence versus absence of others in Study 2 and perceived social closeness in Study 3). Specifically, in the first two studies we used an augmented reality version of a well-validated multisensory interaction task^12^ to measure how PPS boundaries can be shaped by attachment anxiety. In this visuo-tactile detection task participants are asked to respond as fast as possible to a tactile stimulus while attending a task-irrelevant approaching ball using augmented reality. We measured reaction times (RTs) at specific time points, when the ball was perceived at five different distances from the body. Previous studies have shown a facilitation effect (i.e., shorter RTs) when the ball is presented close to the body,^12^ which has been taken as a behavioral proxy of PPS representation. Having a measure of the RTs across five different facilitation distances (from very close to very far from the body, i.e., from D1 to D5, see Figure 1A) allows us to fit a linear function, from which it is possible to extract a regression slope which provides a measure of the differentiation between close (i.e., peripersonal) and far (i.e., extrapersonal) space.^33^^,^^34^ Differently from other measures of PPS, such as the central point of the sigmoid function which provides an in or out zone (i.e., the distance at which the distinction between extrapersonal and peripersonal space is situated^8^^,^^12^^,^^14^^,^^35^), the linear fitting allows us to consider the multisensory response as evolving continuously in space. In fact, a recent account of PPS has challenged the traditional description of an in-or-out space (see^36^ for a review, see also^37^), favoring an interpretation of PPS as a graded field that is best described by a linear function. Thus, even though most of the literature on the social modulation of PPS has focused on an in-or-out space defining a “PPS boundary”, we opted for a measure of less or more differentiation between close and far space, which is more in line with more recent opinions (^36^ e.g., see also^20^ for a recent study using linear slopes), and with more fine-grained modulation of PPS as a function of individual differences.^38^ A reduction in the steepness of the slope indicates that closer and further distances become less distinct, primarily by means of farther distances being treated as if they were nearer in space. In other terms, smaller (i.e., less steep) slopes indicate less differentiation, or a weaker boundary, between peripersonal and extrapersonal space. Despite that “PPS boundary” and “PPS distinction between far and close space” capture different features of PPS representation, the two measures are conceptually related and can provide complementary information. For example, when PPS is less spatially extended, we would expect a steeper slope, meaning a sharper differentiation between peripersonal and extrapersonal space, and vice versa. Thus, based on the existing literature, here we predicted that the higher the attachment anxiety the smaller the slope, particularly in the presence of social support (Study 1) or social observers (Study 2).

Finally, a third, large, preregistered survey study explored the effects of attachment anxiety (ECR-S^39^) on interpersonal space as indexed by reported distance maintained during habitual conversations and the potentially moderating role of current social closeness, as indexed by reported feelings of closeness to other people. We predicted that the higher the attachment anxiety, the closer the preferred interpersonal distance, particularly in close social relations.

## Results

### Study 1: Embodied social support, social context and attachment anxiety on PPS representation

To test whether affective touch as a form embodied social support^40^ that can be optimally manipulated under conditions of bodily threat (for discussion see^40^^,^^41^) modulates the perception of the space surrounding our body, female participants (n = 48; see supplemental information for power calculations) performed the audio-tactile augmented reality interaction task after an experimenter delivered dynamic either slow 3 cm/s CT-optimal touch (affective touch) or very slow 0.3 cm/s non-CT optimal touch (neutral touch condition) on the forearm of participants as a within-subjects manipulation (with the order counterbalanced across participants). Such effects were examined in both a social (i.e., the presence of another individual) and non-social (i.e., the absence of another individual) PPS context as a between-subjects manipulation, i.e., half of the participants were assigned to the social PPS group and the other half to the non-social PPS group (see Figure 1C and STAR Methods for details). Note that we denote ‘social PPS’ to that social context in which there is the mere presence of another individual (e.g., see^42^^,^^43^^,^^44^ for the role of the presence of others versus being alone on behavioral changes), whereas the participant completes the visuo-tactile PPS task. Nevertheless, it is worthwhile noticing that by merely being present, the other person plays a passive role relative to other interpersonal space paradigms (e.g., see^45^^,^^46^). We originally hypothesized peripersonal and extrapersonal space to be less differentiated in the presence of socially supportive cues (similar to other studies where PPS between self and other merge if the other person behaved cooperatively following an economic game^14^), and particularly when that person is around us. In addition, we also examined whether attachment anxiety moderated our effects, given that PPS does not only have action-oriented purposes (e.g., interacting with the environment), but also defensive purposes (e.g., detecting and reacting on threatening stimuli approaching the body^47^) and the perception of social variables themselves depend on individual differences in attachment style,^26^ with the latter modulating bodily threat during social interactions.^41^^,^^48^^,^^49^

**Figure 2:**
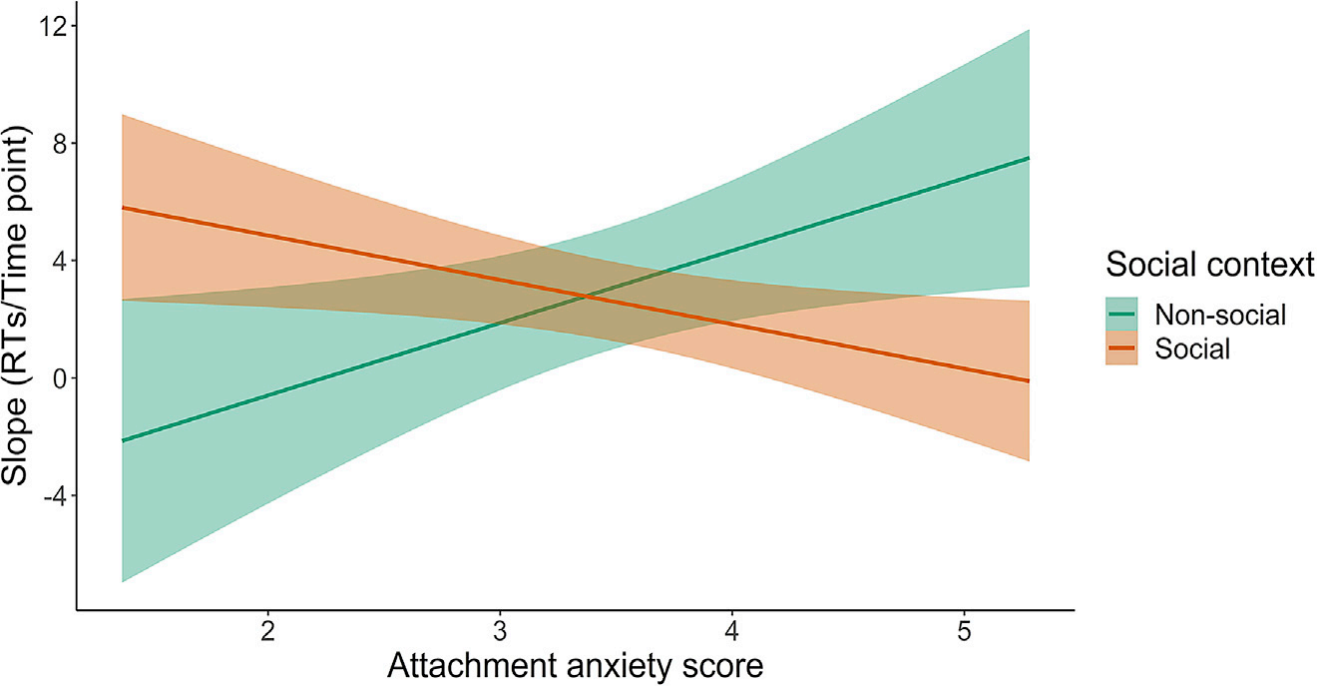
Effects of attachment anxiety and social context on PPS slopes (Study 1) Y axis; slope using a linear function across the five facilitating distances. X axis; attachment anxiety scores. Attachment anxiety scores range from 1 (very low attachment anxiety) to 7 (very high attachment anxiety). In our sample, scores ranged from 1.3 to 5.28. Social PPS and non-social PPS conditions are depicted by the orange and green line, respectively, with 95% shaded confidence intervals. See Figure S2 for individual data points. A steeper PPS slope indicates more differentiation between close and far space. In contrast, a smaller PPS slope indicates less differentiation between close and far space. The difference between social and non-social PPS conditions was significant for low (*b* = 3.96, SE = 1.77, p = 0.025) and high (*b* = -4.76, SE = 1.77, p = 0.007) attachment anxiety. See also Figure S1 for another way to follow up this attachment anxiety by social PPS group interaction, showing the exact same pattern of results.

We first checked that the PPS task and the embodied social support manipulation worked as expected, and then tested the effect of social tactile support on PPS. For the PPS task, baseline-corrected mean RTs to the tactile (vibrating) stimulus administered at the different perceived visual distances were calculated for the approaching ball between the two conditions of the presence versus absence of another person by means of an ANOVA averaging across touch conditions, with factors distance (D1-D5) and social condition group (presence versus absence). As expected from the PPS paradigm,^8^ there was a main effect of distance, *F*(4,184) = 5.72, p<0 .001, η^2^_partial_ = 0.11. Planned comparisons indicate faster RTs in D1, closest distance to the body, relative to D5, farthest distance away from the body, *t*(47) = 3.11, p = 0.003. Even though group (social PPS versus non-social PPS) did not interact with distance, *F*(4,184) = 0.672, p = 0.612, η^2^_partial_ = 0.01, there was a trend for a main effect of group, *F*(1,46) = 3.89, p = 0.055, η^2^_partial_ = 0.08, indicating faster RTs in the non-social versus social PPS group. Given that one would expect faster RTs in response to visuo-tactile stimuli on distances closer to the body, i.e., multisensory boosting effect,^50^ these results indicate that our PPS task was successful. Analyses conducted on the pleasantness ratings scores of both type of touch suggested that slow and very slow touch were perceived as expected in both groups: across groups, participants perceived slow touch (*M* = 76.97, *SD* = 20.65) as significantly more pleasant than very slow touch (*M* = 58.46, *SD* = 19.54), *F*(1,43) = 30.28, p<0 .001, η^2^_partial_ = 0.41. Importantly, group did not interact with touch velocity, *F*(1,43) = 0.44, p = 0.510, η^2^_partial_ = 0.01, indicating that slow touch was perceived as more pleasant than very slow touch, irrespective of the assigned (social, *M*_*slow*_ = 79.50, *SD*_*slow*_ = 19.49; *M*_*veryslow*_ = 63.36, *SD*_*veryslow*_ = 13.62, and non-social, *M*_*slow*_ = 74.76, *SD*_*slow*_ = 21.77; *M*_*veryslow*_ = 54.17, *SD*_*veryslow*_ = 22.98) PPS group. Thus, our social support manipulation induced by affective touch was successful in terms of perceived pleasantness of touch (note that values of three participants were missing because of technical difficulties). Using a linear function (see supplemental information for the equation), the RTs (baseline-corrected) across the five distances were then used to obtain the PPS slope per touch condition (slow versus very slow touch) in both the social and non-social group (see Table S1). Differences between slopes for each condition and group, as well as the moderating role of attachment style, were examined using a linear mixed model. Results suggest that there was no significant main effect of touch condition, PPS group, or their interaction (p’s >0 .275), indicating that slow, supportive touch does not modulate the differentiation between extrapersonal and peripersonal space as compared to slower neutral touch, regardless of the social context. However, we found a significant attachment anxiety by PPS group interaction on PPS slopes, b = 3.75, SE = 1.77, p = 0.034 (see Table S2 for full model results). Post-hoc tests examining differences between conditions, plotted at low (−1 SD), moderate (mean) and high (+1 SD) values of the continuous score of attachment anxiety, showed that the difference between social and non-social PPS conditions was significant for low (b = 3.96, SE = 1.77, p = 0.025) and high (b = -4.76, SE = 1.77, p = 0.007) attachment anxiety, but not for moderate attachment anxiety (b = −0.40, SE = 1.20, p = 0.739); see Figure 2.

### Brief summary of results

The original hypothesis on the role of affective touch in modulating PPS in social context was not confirmed, consistent with previous research that also found no effect of affective versus neutral touch.^51^ However, this first exploratory study indicated that the boundaries between peripersonal and extrapersonal space may depend on the combined effects of social context and attachment anxiety, with people scoring higher in attachment anxiety showing a less defined PPS in the presence of a stranger versus alone in comparison to people with lower scores in this dimension.

### Study 2: High and low attachment anxiety on social and non-social PPS

Given the exploratory nature of the attachment findings in Study 1, we preregistered (https://osf.io/gc5q9) and conducted Study 2 to further investigate the role of attachment anxiety on social and non-social PPS, without any prior administration of tactile stimuli. Furthermore, in Study 1 PPS assessment in social versus non-social context was conducted on a between-subjects basis, given the duration and nature of this study. However, given the finding of individual differences in attachment anxiety on PPS, differences intrinsic to each group, also for the social manipulation, cannot be excluded. Thus, Study 2 employed the social versus non-social PPS condition as a within-subjects factor to better examine the role of attachment style on PPS as a function of the presence of others at the individual level. To specifically examine the effects of attachment anxiety on PPS, a targeted recruitment strategy (see STAR Methods for details) was applied aiming to create two samples (each n = 34) at the two ends of the attachment anxiety distribution, resulting in two groups: high and low attachment anxiety.

**Figure 3:**
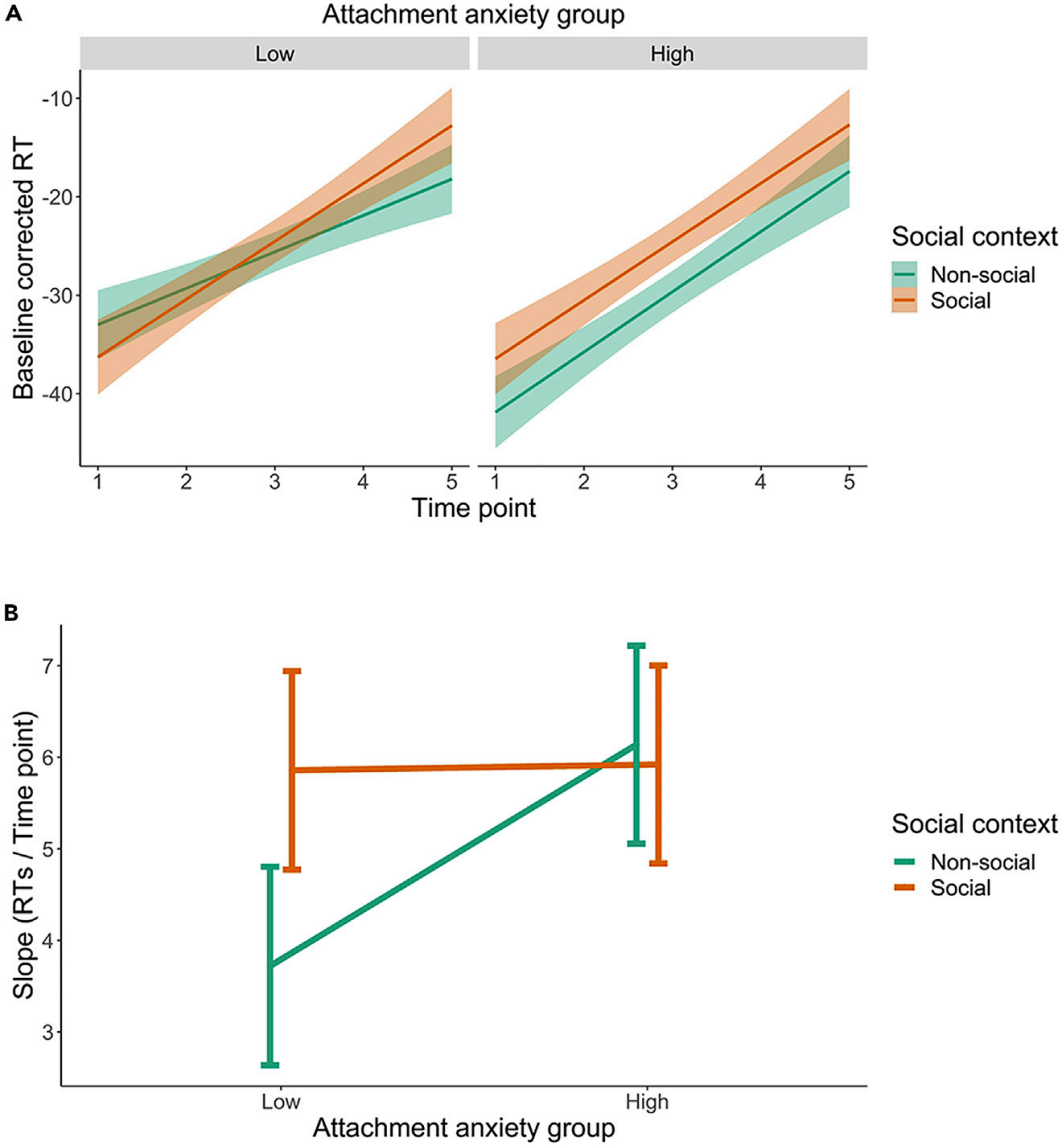
Effects of attachment anxiety and social context on PPS (Study 2) (A) Baseline-corrected RTs across the time/distance points of tactile stimulation in the low (left) and high (right) attachment anxiety groups, as a function of social context. The shading surrounding each line represents the 95% confidence interval. (B) Slope of the baseline corrected RTs over the time/distance points of tactile stimulation, as a function of attachment anxiety groups (Low and High) and Social context (Social – orange and Non-social – green). See Figure S3 for individual data points. The PPS slopes significantly differed between social contexts in the Low attachment anxiety group (*b* = 2.21, SE = 1.03, p = 0.031) and between Attachment anxiety groups in the Non-social context (*b* = 2.55, SE = 0.98, p = 0.009; see Table S6 for full model results). The error bars represent the 95% confidence intervals.

As preregistered, baseline-corrected mean RTs to the tactile (vibrating) stimulus administered at the different perceived visual distances were calculated for the approaching ball. As expected, time/distance point of stimulation had a significant effect on corrected RTs (as a confirmation that the task worked as intended). The closer the ball was to the participant’s face when the tactile stimulation occurred, the faster their responses were (*b* = 5.44, SE = 0.36, p<0 .001). To investigate whether PPS context (presence versus absence of another person) and attachment anxiety influence PPS, from baseline-corrected RTs across the five distances, we computed a linear function to obtain a measure of PPS slope (but see also results for the central point of data fitted sigmoid functions in supplemental information, Figure S4, Tables S7–S9). However, because multilevel modeling is being used in this analysis, instead of considering 2 data points per subject (one slope per each social condition) extracted from average RTs, we decided to include all trials (up to 200 baseline-corrected RT per multimodal trial over both social conditions, in the absence of failed trials – average success rate per run was 95.2%, *SD* = 2.46) to boost analytical power. Note that for transparency and compliance with the preregistration, we present the results obtained using the slopes extracted from average RTs as dependent variable in supplemental information (see Table S3 and S4). Thus, we used baseline-corrected RTs as dependent variable and our targeted interaction between attachment anxiety group, social context and time/distance point as a predictor in the model. As expected from Study 1 results, the 3-way interaction between social context, attachment anxiety and time/distance of stimulation was significant (*b* = −2.38, SE = 1.44, p = 0.048; full model Conditional R^2^ = 0.127, Marginal R^2^ = 0.017); see Table S5. Planned comparisons revealed that this critical 3-way interaction was driven by a higher (steeper) slope in the social condition, as compared to the non-social condition in the low attachment anxiety group, *b* = 2.21, SE = 1.03, p = 0.031, indicating sharper differentiation between peripersonal and extrapersonal space in the presence of a stranger versus when they are alone (Figure 3). However, in people with high scores in the anxious attachment dimension, such differentiation between peripersonal and extrapersonal space remains at high levels even when they are alone, as compared to people with low scores, *b* = 2.55, SE = 0.98, p = 0.009; see Table S6), and does not change depending on the social context (*b* = −0.17, SE = 1.02,p = 0.869; see Table S6). For transparency, the use of baseline corrected RTs as dependent variable was not preregistered and as such this analysis is considered exploratory. Of note, however, the preregistered results using slopes as dependent variable, even though not statistically significant, mirror the exploratory results.

### Brief summary of results

Our hypothesis on people scoring higher in attachment anxiety showing a less defined PPS in the presence of a stranger versus alone was not confirmed. Although we found in our exploratory analysis that attachment anxiety still modulated the differentiation between peripersonal and extrapersonal space depending on the social context, this was observed in a somewhat different way than expected. Specifically, we found that in people with high attachment anxiety scores, PPS does not change based on social context as it does in people with low attachment anxiety scores. Instead, in people with high anxiety scores, the differentiation between peripersonal and extrapersonal space remains constantly sharp irrespective of the social context.

### Study 3: Interpersonal distance on perceived distance in conversations

To investigate whether individual differences in attachment anxiety also shape the preferred interpersonal distance that people feel comfortable maintaining during a conversation, an index of their ‘interpersonal space’, we recruited participants through a larger national survey conducted with collaborators with a broader scientific scope (see STAR Methods for details). Based on our inclusion and exclusion criteria, our final sample for this study consisted of 19,417 participants (as preregistered: https://osf.io/g7h58; see also supplemental information for inclusion criteria).

**Figure 4:**
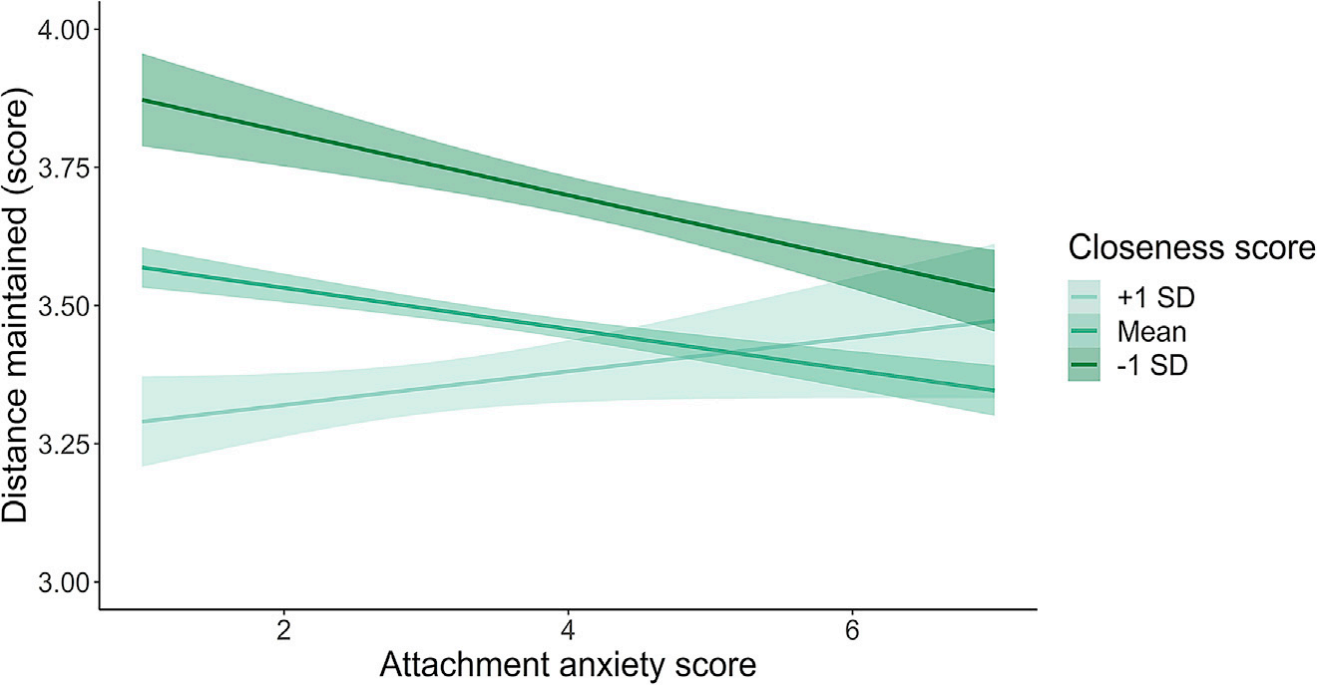
Effects of attachment anxiety on distance maintained during a conversation based on feelings of closeness (Study 3) The main effect of attachment anxiety over reported distance maintained during a conversation was statistically significant (*b* =0 .11, SE = 0.01, p<0 .001, Marginal R^2^_(diff)_ = 0.018) and was modulated by feelings of closeness (*b* = 0.01, SE = 0.01, p = 0.010, Marginal R^2^_(diff)_ = 0.001). The shading surrounding each line represents the 95% confidence interval.

In the preregistration, we hypothesized that individuals with higher attachment anxiety scores would report less distance maintained during a conversation only when attachment avoidance scores were also relatively low. We expected such results to depend on low attachment avoidance because if such dimension was also high, this could have canceled our results given the opposite nature of the two attachment dimensions. Moreover, such moderation of effects by attachment avoidance is likely to play a role when it comes to an explicit, self-reported question about comfort in social relations, relative to a more implicit objective measure such as PPS. Our results, however, indicate that although the effect of attachment anxiety on interpersonal space was as predicted, i.e., the higher the anxiety the less the reported distance maintained (*b* = −0.04, SE < 0 .01, Marginal R^2^_(diff)_ = 0.002, p<0 .001), this effect was not dependent on the attachment avoidance score (results showed no modulatory effect; *b*< −0.00, p = 0.482). Furthermore, although the main analysis in this study was verifying the effect of attachment style on interpersonal space as manifested by reported distance maintained during a conversation (as above), in a stepwise manner, as preregistered, we also checked for possible modulation effects of current social closeness and developmental touch history (as well as personality and interoceptive self-efficacy on secondary analyses, see Table S11 and Table S12) on such effects (ethnicity, sexuality, and date when the survey was completed were added as random effects and age was added as a covariate in the full model as preregistered). Although we did not expect attachment anxiety to be moderated by developmental touch history (too much variability/variance between individuals), we expected it to be moderated by social closeness, in that the more the attachment anxiety, the closer the preferred interpersonal distance particularly when they report feeling less close to others in general. When adding closeness and developmental touch history to the model and their interactions with attachment style, statistically significant modulatory effects of social closeness were found (for full model results see Table S10). Even though the main effect of attachment anxiety on interpersonal space did not change, closeness moderates the amplitude of these effects (*b* = 0.01, SE = 0.01, p = 0.010, Marginal R^2^_(diff)_ = 0.001; see Figure 4). The lower the score in attachment anxiety the less the distance people report in conversion only in people who report feeling the closest to others, relative to those who report not feeling close to others, whereas the higher the score in attachment anxiety, the less the distance irrespective of closeness.

### Brief summary of results

Consistent with our predictions, we found that the higher the attachment anxiety, the closer the preferred interpersonal distance. However, although this effect was moderated by people’s feelings of social closeness, the pattern of effects was different from what was predicted and more similar to the results of Study 2, namely people scoring high in attachment anxiety preferred closer interpersonal interactions and this preference were less affected by how close they felt to others, whereas the comfort of social distance in people with less attachment anxiety appeared to vary depending on their feelings of social closeness.

## Discussion

We studied how attachment anxiety affects our social peripersonal (PPS) and interpersonal (preferred interpersonal distance) space. First, in an exploratory study, we tested whether embodied social support (by using affective versus neutral touch^40^) and social observers, modulate the perception of the space surrounding our body, while also secondarily exploring the role of attachment anxiety. Although we found no evidence of change in PPS after affective touch as compared to neutral touch (as in^51^), or that such effects were moderated by attachment anxiety, we found that the differentiation between peripersonal and extrapersonal space depended on attachment anxiety and the particular social context. To further investigate this effect, and to exclude that it depended on our tactile manipulations, we conducted a follow-up, preregistered study assessing PPS representation as a function of individual differences in attachment anxiety. We found that attachment anxiety still modulated the differentiation between peripersonal and extrapersonal space depending on the social context but in a somewhat different way than expected. Specifically, people scoring high in attachment anxiety, relative to people scoring low in attachment anxiety, demonstrate differentiation between peripersonal and extrapersonal space, which does not change depending on the social context; in contrast, in people scoring low in attachment anxiety – i.e., those more securely attached – the separation between PPS and extrapersonal space appears as more flexible, in that it changes as a function on social context. In a final, preregistered, large-scale survey of the UK population, we investigated whether attachment anxiety also impacted on the interpersonal space people felt comfortable with during habitual conversion and its relation with social closeness. As predicted, we found that people with higher attachment anxiety prefer to stand closer to others during conversation, but this tendency was not modulated by feelings of social closeness, as it was for people with less social attachment anxiety. Taken together, our studies suggest that attachment anxiety reduces the flexibility of peripersonal and interpersonal space, particularly during social settings. These findings are discussed in detail below.

Given that attachment anxiety is typically associated with craving constant closeness,^27^ we expected that the higher the attachment anxiety, the less defined the PPS in social settings characterized by embodied social support, particularly if we consider PPS as a critical space for triggering approaching and defensive behaviors.^9^ Although this original hypothesis was not confirmed in Study 2, we found that the differentiation between peripersonal and extrapersonal space depends on the social context and attachment anxiety. Our new finding suggests that when people scoring lower in attachment anxiety are alone, they do not differentiate peripersonal and extrapersonal space as sharply as they do when in the presence of a stranger. Presumably because the gradient of differentiation between their own and other’s space becomes sharper as they do not yet know this person and/or their intentions.^14^ Conversely, when people scoring higher in attachment anxiety are in the presence of a stranger, relative to alone, their differentiation between peripersonal and extrapersonal space becomes less defined. This finding is in line with research suggesting that the hyperactivation of the PPS parietal frontal cortical network seems to be specific to social cues^25^ and can be explained by these individuals’ constant mistrust and persistent worrying and checking for signals of support from the environment.^27^ Of interest, another line of work proposed that PPS expands by trait-anxiety^23^ in line with the idea that the functioning of the human defensive systems relates to personality traits such as fear and anxiety.^52^ That is, the safety margin in anxious individuals is located at a further distance from their bodies. Here, we extend this line of research by showing that this effect plays a specific role in the social domain. Indeed, our effects are likely because of our measure (ECR-R) tapping into individual differences in anxiety in the context of attachment relationships. However, our findings regarding attachment anxiety were exploratory and we cannot exclude with certainty that being touched did not have any effect on our findings regarding attachment anxiety. Instead, we can only say that attachment anxiety modulated the differentiation between personal and extrapersonal space in opposite ways in social and non-social contexts, irrespective of tactile stimulation. Moreover, our social versus non-social manipulation was conducted on a between-subjects basis (deemed necessary given the duration and nature of this study). Therefore, differences intrinsic to each group cannot be excluded.

Thus, in Study 2 we focused on the role of attachment anxiety on the social modulation of PPS, without any prior administration of tactile stimuli, and with the social versus non-social PPS condition as a within-subjects factor to better examine the role of attachment style on PPS. We expected that the higher the attachment anxiety the smaller the slope, indicating a less defined PPS, particularly in the presence of social observers (as found in Study 1). However, such hypothesis was not confirmed. Even though we found that people with low scores in the attachment anxiety dimension regulate their spatial representations as a function of social context, so that differentiation between peripersonal and extrapersonal space is sharper when in the presence of a stranger than when alone (as in Study 1 and as in^14^), our findings on people with high scores in attachment anxiety were somewhat different from those predicted and observed in Study 1. Surprisingly, in our exploratory analysis, we found that peripersonal-extrapersonal differentiation remains at high levels in people with high scores in the anxious attachment dimension even in the absence of a stranger. It is thought that PPS boundaries act as a sort of defensive bubble surrounding the body that changes not only according to the emotional content of the stimulus approaching the body,^53^ but also according to individual characteristics of the observers (e.g.,^20^^,^^21^ but see also^23^^,^^54^^,^^55^ for similar results, although note that a different paradigm was used in the latter) and the interpretation of the overall safety of a situation.^53^ It therefore appears that in people with high levels of attachment anxiety this malleability of the PPS is reduced, and their PPS remains rigidly more segregated from the extrapersonal space. One interpretation for this effect is that this reduced malleability and sharp distinction between personal and extrapersonal space observed in people with anxious attachment is related to their documented social hypervigilance, checking for signs of support and persistent worrying about rejection or, abandonment, as also reflected in a hyperactive PPS monitoring brain network.^25^

Turning now to our final, third study, where we examined, as preregistered, the effects of individual differences such as attachment anxiety on interpersonal space^29^ and we found that the higher people scored on attachment anxiety, the closer the distance they preferred to maintain during a conversation. These results are consistent with previous research suggesting that attachment anxiety is positively correlated with interpersonal distance when another participant approaches a stationary participant^31^^,^^56^ and that adolescents higher in anxious ambivalent attachment let others intrude into their personal space to an uncomfortable degree, probably because of fears of being rejected.^57^ As preregistered we also explored the modulatory role of current social closeness and developmental touch history. Of interest, we find that current social closeness (i.e., how close people have been feeling toward other people, see STAR Methods for details) moderates these effects. Specifically, people scoring high in the attachment anxiety dimension seem to differentiate their interpersonal distance less based on feelings of social closeness. Given the need for people scoring high in attachment anxiety for close proximity,^27^ they may need less distance, or more closeness, during conversations, no matter how close they have been feeling toward other people. This reduced malleability of preferred interpersonal distance in the face of different social contexts is compatible with similar findings on peripersonal space in Study 2. Taken together, our first exploratory study on the relationship between attachment, touch and PPS, and then in a more focused way, our two preregistered studies suggest that during interpersonal interactions attachment anxiety reduces the flexibility of different facets of our perceived personal space, including both peripersonal and interpersonal space. These effects possibly relate to the increased social hypervigilance and rejection insecurity associated with high attachment anxiety, leading individuals not to adjust their perceived personal space depending on social context, as for example seen in people with autism spectrum disorder.^58^ Instead, these individuals appear to experience the space around the body as though they are never securely surrounded by others and to seek social proximity with others irrespective of social closeness. Given the developmental nature of attachment style and particularly attachment anxiety^24^^,^^27^ according to attachment theory we speculate that such hypervigilant strategy developed in early childhood for adaptive reasons, in response to deficits in the caregiver’s responsiveness to the infant’s needs.

More generally, these findings also highlight how the cognitive mental representations and high-level social processes that are associated with individual differences in adult attachment styles may also extend to certain unconscious, multisensory processes and perceptual experiences of the space around the body^59^^,^^60^ in the social world.

Despite these insights, our findings should be interpreted in light of their limitations and future directions. First, future studies could substantiate these interpretations by manipulating social rejection before assessing the social modulation of PPS or interpersonal space. Second, our sample in Study 1 and 2 consisted of women only and future studies should examine whether the results on PPS extend to men. Third, recent findings have shown that proximity to the body alone does not determine PPS. In fact, other factors not related to the stimulus position (e.g., walking, vestibular cues, stimulus direction, trajectory, valence and semantics, or even the landscape) have been found to also shape PPS.^36^ Thus, future studies should examine the effects of attachment anxiety on PPS using other factors other than proximity. Finally, here we bridged interdisciplinary fields ranging from interpersonal aspects in social psychology to PPS in cognitive science. Even though this interdisciplinary bridge has its advantages (e.g., deeper understanding, wider audience, etc.), there are certain limitations that should be acknowledged. For instance, although related^13^^,^^28^^,^^29^^,^^61^ interpersonal distance and PPS are different constructs with different methodologies,^13^ as also evident from different methodologies embedded in this study. Future research is still needed to fully elucidate their complex relationship.

In sum, we conclude that attachment anxiety reduces the social malleability of both peripersonal and interpersonal space.

## STAR★Methods

### Key resources table

**Table:** 

REAGENT or RESOURCE	SOURCE	IDENTIFIER
**Deposited data**		

Raw and analysed data Study 1	This paper	https://doi.org/10.17605/OSF.IO/TU4V9
Raw and analysed data Study 2	This paper	https://doi.org/10.17605/OSF.IO/TU4V9
Raw and analysed data Study 3	This paper	https://doi.org/10.17605/OSF.IO/TU4V9

**Other**		

Analyses code study 1	This paper	https://doi.org/10.17605/OSF.IO/TU4V9
Analyses code study 2	This paper	https://doi.org/10.17605/OSF.IO/TU4V9
Analyses code study 3	This paper	https://doi.org/10.17605/OSF.IO/TU4V9

### Resource availability

#### Lead contact

Further information and requests for resources and reagents should be directed to and will be fulfilled by the lead contact, Mariana von Mohr (mariana.vonmohr@rhul.ac.uk).

#### Materials availability

This study did not generate new unique reagents.

### Experimental model and subject details

#### Participants

We recruited 48 healthy females for study 1 (Age: M = 28.87.3, SD = 3.29), 68 healthy females for study 2 (Age: M = 23.26, SD = 3.44) and 19,417 healthy females and males for study 3 (Age: M = 57.3, SD = 13.77). Only females were recruited for Studies 1 and 2 to control for gender effects related to touch^62^^,^^63^ and the gender of the person sitting in front of the participant in the social PPS condition, who was also female. All subjects gave their informed consent to participate in this study, which was approved by appropriate institutional Ethics Committees at University College London (UCL) (study 1 and 2) and Goldsmiths, University of London (study 3).

Study 3 data originated from a large, national touch survey organised by the Wellcome Trust in collaboration with Goldsmiths (University of London), University College London and the British Broadcasting Corporation. Inclusion criteria consisted of participants living in the United Kingdom, being 18 yearsold or older, and having a valid subscale score in both attachment anxiety and attachment avoidance. The survey was launched on the 21.01.2020 and closed on the 30.03.2020.

The studies were run in accordance with the declaration of Helsinki.

#### Power calculations

Study 1. Due to difficulties in conducting a power analysis on a multilevel model, the sample size was selected based on prior F-tests calculations (f(U) set at 0.453, within-between interaction, with alpha = 0.05 and power = 0.80, G power 3.1) in accordance with an effect size reported in similar studies (η2partial = 0.06; Pellencin et al., 2018) examining the influence of social perception on PPS.

Study 2. The sample size was estimated based on an effect size of d = 0.3, calculated on the basis of prior research with a similar design and MLM analysis (study 1). Using G∗Power 3.1 software with power (1-beta) set to 80%, alpha set to 0.05%, and an effect size of d = 0.3, the required sample size was 64 participants.

### Method details

#### PPS task

##### Apparatus and stimuli

The PPS task was administered using a virtual reality headset (Oculus Rift DK2; 900 × 1090 per eye, ∼105 FOV) and the ExpyVR software (https://lnco.epfl.ch/expyvr): a new augmented-reality technology developed at the EPFL. (Laboratory of Cognitive Neuroscience at the Ecole Polytechnique Federale deLausanne). During each trial, a looming ball on a transparent background was presented, while in approximately 77% of the trials, subjects also received mild (non-painful) vibrations on their left-hand fingertips by means of holding sensory electrodes (i.e., vibrotactile stimulator, custom-made at the EPFL). The remaining trials were catch trials, where only the looming ball was presented. Subjects were asked to respond to the vibration stimulation as fast as possible, by pressing the space bar on the keyboard. Tactile RTs were recorded.

For experiment 1 and 2: in the social PPS task condition, an experimenter was sitting in front of the participants (90 cm away) with the looming ball appearing at the level of the neck of the experimenter. In contrast, in the non-social PPS condition, there was no person sitting in front of the participant, but the chair was still present.

#### Tactile manipulation (study 1)

In experiment 1, a skin area (9 × 4 cm) was marked on the participant’s left forearm (i.e., stimulation site). While the only other study investigating the role of affective touch on PPS used skin-to-skin touch,^51^ here we used a soft make-up brush to deliver the slow, affective and very slow, neutral touch. On the one hand, using a soft brush to deliver the touch, as compared to skin-to-skin contact, allows us greater experimental control over confounding factors such as differences in skin temperature, sweating rates, etc. On the other hand, however, it remains possible that brush stroking may have missed essential mechanisms of everyday skin-to-skin socio-tactile interactions. Interestingly, the two types of touch have been shown to be perceived as equally pleasant when delivered through skin-to-skin contact,^51^ which as proposed by the original authors, could be responsible for the absence of a significant difference between affective and neutral touch on PPS. However note that in the current study affective touch is perceived as more pleasant than neutral, yet we also found that type of touch did not have an effect on PPS.

Participants were told that they would be receiving touch from the experimenter using a make-up brush on their left forearm, followed by the PPS task in which they would observe a looming ball and receive some tactile stimuli from the electrode they were holding on their left hand. They were asked to respond to the tactile vibrating stimuli as fast as possible by pressing the space bar and to ignore the visual stimuli presented on the head-mounted display when responding. The experiment consisted of two blocks. In each block, participants received one of the two stroking velocity conditions (slow or very slow touch) from the same experimenter (with the order of the stroking velocity conditions counterbalanced across participants). Each block was divided into 10 mini-blocks, where participants first received 1 min of touch while keeping their eyes closed, followed by 18 PPS trials (see Figure 1C and below for the PPS trials).

#### Peripersonal space measurement

The PPS task included three types of trials, namely bimodal visuo-tactile, unimodal tactile and catch trials. The critical bimodal visuo-tactile trials started with the appearance of the ball on the center of the transparent screen, gradually approaching the participants for approximately 2600 msec. Together with the visual stimulus, a tactile stimulation (lasting 200 msec) was delivered to the participant’s left hand by the vibrotactile stimulator. The tactile stimulator was given at 5 temporal delays from the appearance of the ball (after 433, 866, 1299, 1732, 2165 msec, with the first corresponding to Time 5 and the last to Time 1) and consequently, perceived by the participant when the virtual, visual object was placed at 5 different distances from her (from very close to the body, Distance 1, to very far, Distance 5). In this sense, the longer delay corresponds to a closer distance. In other words, 433 msec from the beginning of the movement of the ball (Time 5) would correspond to the farthest distance perceived by the participant, in this case D5 – and vice versa. In the second type of trial, namely unimodal tactile and unimodal visual/catch trials, participants received the tactile stimuli at the same time intervals but no ball was presented or the ball was presented without any tactile stimuli respectively. The visual unimodal trials are included as a manipulation check to make sure the participants are not giving any responses by pressing the space bar. In contrast, the unimodal tactile serves as a baseline for how quickly participants respond to the tactile stimuli without any visual stimuli, i.e., to control for the expectancy effect of temporal delay of tactile stimulation on the subject’s response (see Figures S6 and S7 for confirmation that the paradigm worked for Study 1 and 2, respectively).

Each PPS block consisted of 180 trials: 30 unimodal visual trials, 50 unimodal tactile trials (10 for each distance), and 100 bimodal trials (20 trials for each distance) divided into 10 mini-blocks and presented in a pseudorandom order with an interstimulus interval of 0.9, 1.15, 1.4, 1.65 and 1.9 s. RTs were then inspected and trails where RTs were above or below 2.5 SD of the RT mean of the participant were removed. The average percentage of success (or not fails), including trials in which the participant failed to give a response, was 94.88 (SD = 2.98) for study 1 and 95.2% (SD = 2.46) for study 2. Tactile unimodal and visuo-tactile stimuli RTs were then averaged separately across distance in each PPS block for each participant. Using a conservative approach, reaction times (RTs) from each distance were then baseline corrected by subtracting the unimodal tactile responses (msec) from the visuo-tactile responses (msec) (e.g., as done in.^12^^,^^14^ This delivered RTs (baseline corrected) across five distances for each participant per touch condition.

##### PPS slope equation

The slope of the latter was extracted using a linear function (MatLab 2015b), which reflects the amount of segregation between the close (peripersonal) and the far (extrapersonal) space. The linear function was described by the following equation: y(x) = yo + k∗x, where x represents the timing of tactile delivery in ms (independent variable), y the reaction time (dependent variable), yo the intercept at x = 0 and k is the slope of the linear function.^12^ Thus, a ‘PPS slope’ was obtained for each participant per touch condition (slow vs. very slow touch) in both the social and non-social group. A steeper, or bigger, ‘PPS slope’ indicates more differentiation between close and far space. In contrast, a smaller ‘PPS slope’ indicates less differentiation between close and far space. That is, a reduction in the slope would indicate that closer and further distances become less distinct, primarily by means of farther distances being treated as if they were nearer in space.

As preregistered, in Study 2 we also fitted a sigmoid function to the data in order to obtain a measurement of the PPS boundary through the central point of the sigmoid (as in^12^) and conducted exploratory analyses; see Figure S4 and Table S7 for these results.

#### Interpersonal distance

In experiment 3 we used a question reporting how close participants stand when talking to someone: “I stand very close (for example, less than 1 m away) when talking to someone” (measured on a 5-point scale from “Strongly agree” to “Strongly disagree”, to measure interpersonal distance).

#### Current social closeness

In experiment 3 we used one question reporting how close people have been feeling toward other people: “I’ve been feeling close to other people” (measured on a subjective 5-point scale from “None of the time = 1” to “All of the time = 5”).

#### Attachment anxiety

In experiments 1 and 2 we used the ECR-R^64^ questionnaire to measure adult attachment style. This 36-item (7-point scale) questionnaire is well-validated^27^ and measures individual differences with respect to the extent to which individuals are insecure about the responsiveness and availability of close others (i.e., attachment anxiety) and the extent to which individuals are uncomfortable with being close and depending on close others (i.e., attachment avoidance). The ECR-R attachment anxiety subscale was used as a continuous predictor variable in Experiment 1 (scores ranging from 1.3 to 5.28; *M* = 3.51, *SD* = 1.03 in the social PPS group; and *M* = 3.41, *SD* = 1.02 in the non-social PPS group, note there were no differences in attachment anxiety between the groups, t(46) = 0.338, p = 0.737) and for participants selection in Experiment 2. As pre-registered, the ‘Low Attachment Anxiety’ group comprised participants scoring below the 25th percentile of the population ECR-R attachment anxiety sub-scale score (Attachment anxiety: *M* = 1.86, *SD* = 0.59; Attachment avoidance: *M* = 2.31, *SD* = 0.77), and the High group by participants above the 60^th^ percentile (Attachment anxiety: *M* = 4.62, *SD* = 0.65; Attachment avoidance: *M* = 3.41, *SD* = 1.16) (note that, as pre-registered, following a low initial recruitment rate, the high percentile cut-off was reduced from 75th to 60^th^ percentile). Half of the recruited participants (N = 34) were in the “Low” group (Age: *M* = 24.21, *SD* = 3.90) and the other half in the “High” group (Age: *M* = 22.32, *SD* = 2.65), with regards to attachment anxiety score.

In experiment 3 we instead employed the ECR-S, a shorter version of the well-known Experience in Close Relationship Scale (ECR-R^39^) comprising 12 items. This was due to being part of a larger survey and in order to reduce the overall survey length.

#### Other measures

We used other self-reported measures such as general anxiety (STAI^65^), perceived trustworthiness (General Trust Scale^66^) and social status (MacArthur Scale of Subjective Social Status^67^), to see if any of the observed effects in peripersonal space were explained by these variables (study 2). Similarly, in experiment 3, the effects of attachment style, personality (Big Five Inventory-ShortForm^68^) and interoceptive self-efficacy (Interoceptive Accuracy Scale; IAS^69^) over interpersonal space, considering people’s developmental touch history (2 top loading items composite of the subscale childhood touch of Touch Experiences and Attitudes Questionnaire^70^) and perceived current social closeness (one question reporting how close people have been feeling towards other people, measured on a subjective 5-point scale from “None of the time” to “All of the time”), were used to check the possible modulation effects of these variables in a stepwise manner, to see if these variables further modulated interpersonal distance (and its interaction with attachment anxiety). These results can be found in supplemental information, e.g., Tables S8–S12, Figure S5).

### Quantification and statistical analyses

Statistical analyses were conducted in STATA (version 15) and R/R Studio. Statistical significance is defined as p <0 .05. *Study 1*. Using a linear function, the RTs (baseline corrected) across the five distances were used to obtain the PPS slope per touch condition in both the social and non-social group (see Table S1). Differences between slopes for each condition and group, as well as the moderating role of attachment style, were examined using a linear mixed model. For our outcome variable (PPS slope, extracted using linear fitting) in each group, we specified multilevel models with touch condition (slow touch/very slow touch) and PPS group (social/non-social) as dummy-coded categorical predictors. To assess the role of attachment anxiety, we first specified attachment anxiety as a continuous predictor in our model, and included all interaction terms, while controlling for attachment avoidance (although note that we obtain the exact same pattern of results with and without attachment avoidance in the model). In Table S2 we present the full model results. The significant interaction between attachment anxiety and PPS group (social vs non-social) over PPS slopes is highlighted. See also Figure S8 for analyses conducted on the RTs instead of the PPS slope, i.e., databefore fitting, showing a similar pattern of results, namely a significant interaction between distance, attachment anxiety and PPS group. *Study 2*. In a similar faction to study 1, a linear function was used to obtain a PPS slope across the five distances per social condition per participant. In Table S3 we present the mean baseline-corrected reaction times at each distance and the corresponding mean PPS slope for each attachment group and social condition. Differences between slopes for each condition and group were examined using a linear mixed model. However, the main results presented above reflect analysis done using baseline corrected RTs as dependent variable (as to not collapse data points). The final model, testing the 3-way interaction of interest, included attachment anxiety group, time point of stimulation, social context, and their interactions as independent variables. In Table S4 and Table S5 we present the full model results corresponding to using the PPS slope as dependent variable and the baseline corrected RTs as dependent variable, respectively. Note that we obtain the exact same pattern of results when controlling for attachment avoidance continuous scores in the latter model (see Table S5). *Study 3.* We used step-wise multilevel modeling (MMLM) as preregistered to examine our predicted effects. In our base model we used time the survey was completed, as well as demographic variables as random effects. Random effects that explained less than 0.01 of the variance in the model (ICC <0.01) were removed to enhance parsimony of the model. We first included attachment anxiety, attachment avoidance and their interaction as predictors, while controlling for developmental touch history and closeness (see Table S10). In a stepwise manner, we also proceeded to check possible modulation effects of current social closeness, and developmental touch history. Next, as preregistered, secondary exploratory analyses investigated the effects of interoception self-efficacy (as measured by the Interoceptive Accuracy Scale) and personality (as measured by the Big Five Inventory-ShortForm) on interpersonal space, as well as its modulation of the effects found of attachment style over interpersonal space. The full model results of these analyses can be found in Table S11 (regarding interoceptive self-efficacy) and Table S12 (regarding personality). All continuous predictors, in all three studies, were mean-centred in order to avoid multicollinearity issues.

## Data Availability

• All original data and code have been deposited at OSF (https://osf.io/tu4v9/) and are publicly available as of the date of publication. DOIs are listed in the key resources table. • Any additional information required to reanalyze the data reported in this paper is available from the lead contact upon request.
