# Supplementary material for: My social comfort zone: Attachment anxiety shapes peripersonal and interpersonal space
Source: iScience. 2023 Jan 11;26(2):105955. doi: 10.1016/j.isci.2023.105955 (PMC9883291; doi:10.1016/j.isci.2023.105955)
Supplement: Document S1. Figures S1–S8 and Tables S1–S12 [file mmc1.pdf]

iScience, Volume *26*

Supplemental information

My social comfort zone: Attachment anxiety

shapes peripersonal and interpersonal space

Mariana von Mohr, Paulo C. Silva, Eleonora Vagnoni, Angelika Bracher, Tommaso Bertoni, Andrea Serino, Michael J. Banissy, Paul M. Jenkinson, and Aikaterini Fotopoulou

Supplementary Materials

Table S1. Mean (SD) for RTs (msec; baseline corrected) at each distance and extracted PPS slope for each touch condition and social group, related to STAR Methods.

	D1	D2	D3	D4	D5	PPS slope
Social PPS						
Slow touch	-20.52 (4.51)	-25.85 (4.31)	-12.64 (2.86)	-17.08 (4.55)	-15.06 (4.25)	1.97 (4.57)
Very slow touch	-19.96 (4.17)	-27.97 (3.36)	-19.16 (5.19)	-9.28 (4.28)	-13.41 (4.44)	3.18 (5.14)
Non-social PPS						
Slow touch	-35.42 (5.09)	-25.98 (6.34)	-31.50 (6.70)	-17.86 (4.93)	-21.11 (4.99)	3.67 (7.68)
Very slow touch	-25.99 (5.05)	-34.61 (6.69)	-24.90 (4.86)	-22.52 (5.71)	-20.68 (4.35)	2.27 (2.27)

Table S2. Full multilevel model results for PPS slopes, controlling for attachment avoidance, related to STAR Methods.

Effect	b	*SE*	*p*-value	Confidence intervals Lower	Upper
Slow vs. very slow touch	1.21	1.69	.474	-2.11	4.52
Social vs. non-social PPS group	1.70	1.69	.314	-1.61	5.02
Touch condition * PPS group	-2.61	2.39	.275	-7.30	2.07
Attachment anxiety	-1.62	1.19	.174	-3.96	.72
Touch condition * attachment anxiety	.12	1.68	.942	-3.18	3.42
**PPS group* attachment anxiety**	**3.75**	**1.77**	**.034**	**.29**	**7.22**
Touch condition *PPS group * attachment anxiety	1.16	2.39	.626	-3.53	5.86
c. Attachment avoidance	-.45	.65	.485	-1.73	.82

Note. Mean adult attachment styles were M = 3.51 (SD= 1.03) for attachment anxiety and M = 3.51 (SD = .75) for attachment avoidance in the social PPS group, and M = 3.41 (SD= 1.02) for attachment anxiety and M = 2.92 (SD = 1.31) for attachment avoidance in the non-social PPS group. There were no significant differences in attachment anxiety, or attachment avoidance, between the social and non-social PPS group, t(46)=.338, p=.737, t(46)=1.929, p=.060, respectively (there were also no significant differences in attachment anxiety or avoidance between the current samples and the general population). Across the groups, attachment anxiety and attachment avoidance were not correlated, although a trend was observed, r = .26, p=.059. Note that we get the exact same pattern of results if we do not include attachment avoidance in the model.

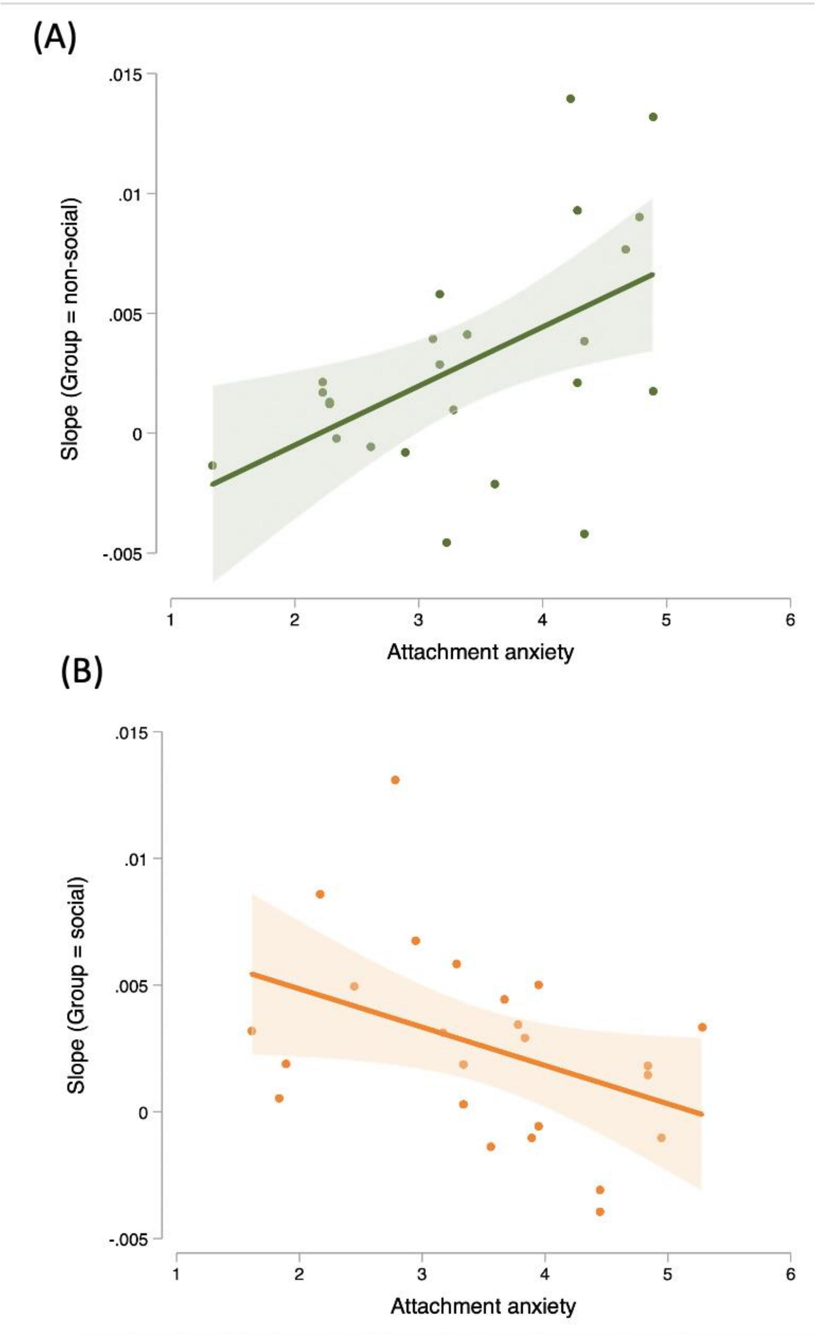

Figure S1. Correlations between attachment anxiety scores and slopes in the non-social and social condition, related to Figure 2. (A) attachment anxiety correlates with slope in non-social

group r=.52, p=.009. (B) Attachment anxiety correlates with slope in social group= -.41, p=.047.

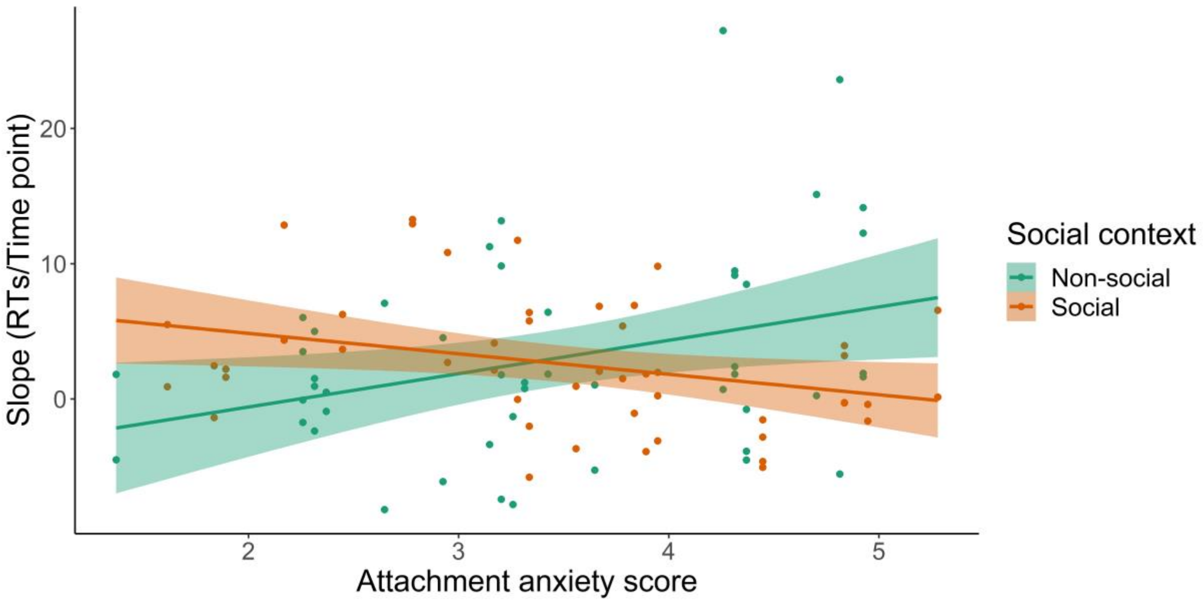

Figure S2. Effects of attachment anxiety on PPS slopes. Y axis; slope using a linear function across the five facilitating distances. X axis; attachment anxiety scores, related to Figure 2. Social PPS and non-social PPS conditions are depicted by the blue and grey line, respectively, with 95% shaded confidence intervals. A steeper PPS slope indicates more differentiation between close and far space. Small circles indicate individual data point.

Table S3. Mean (SD) for RTs (msec; baseline corrected) at each distance and extracted PPS slope for each attachment anxiety group and social condition, related to STAR Methods.

	D1	D2	D3	D4	D5	PPS slope
High Group						
Social	-39.03 (29.52)	-29.03 (31.00)	-22.47 (22.84)	-15.97 (26.01)	-16.02 (32.80)	5.91 (9.10)
Non-social	-40.87 (34.53)	-37.58 (28.64)	-29.69 (26.34)	-22.87 (31.87)	-17.35 (29.68)	6.18 (7.09)
Low Group						
Social	-36.53 (42.02)	-32.38 (26.27)	-26.41 (25.09)	-9.08 (27.05)	-19.10 (34.79)	5.82 (9.05)
Non-social	-30.92 (28.51)	-31.52 (32.61)	-26.51 (20.18)	-19.60 (30.51)	-18.02 (38.70)	3.77 (8.35)

Table S4. Full multilevel model results using PPS slopes as dependent variable, related to STAR Methods.

Effect	b	*SE*	*p*-value	Confidence intervals	
				Lower	Upper
Attachment anxiety group (High - Low)	2.41	2.02	.232	-1.54	6.36
Social context (Social - Non-social)	2.05	1.69	.224	-1.26	5.35
Group X Social context	-2.32	2.39	.331	-6.99	2.36

Table S5. Multilevel results using Baseline corrected RT's as dependent variable in the step-wise model building, related to STAR Methods.

Effect	b	*SE*	*p*-value	Confidence intervals	
				Lower	Upper
Attachment Anxiety group (High - Low)	-2.00	4.53	.659	-10.87	6.88
**Social context (Social - Non-social)**	**3.03**	**1.03**	**.003**	**1.02**	**5.04**
**Time point of stimulation**	**5.44**	**.36**	**< .001**	**4.74**	**6.15**
**Attachment anxiety group * Social context**	**4.28**	**2.05**	**.037**	**.26**	**8.30**
**Attachment anxiety group * Social context * Time point of stimulation**	**-2.38**	**1.44**	**.048**	**-5.21**	**0.45**

Note. Including attachment avoidance as a covariate, b=-3.02, SE=2.30, p=.192, yielded the exact same pattern of results, and our attachment anxiety group by social context by time point of stimulation remained statistically significant, p=.048.

Table S6. Post-hoc test results on the 3-way interaction between social context, attachment anxiety and time point of stimulation, related to Figure 3.

Effect	b	*SE*	*p*-value	Confidence intervals	
				Lower	Upper
High Group (Social context X Time point)	-.17	1.02	.869	-2.17	1.83
**Low Group (Social context X Time point)**	**2.21**	**1.03**	**.031**	**.20**	**4.21**
Social (Group X Time point)	.16	1.04	.879	-1.87	2.19
**Non-social (Group X Time point)**	**2.55**	**.98**	**.009**	**.63**	**4.48**

Note. The first two rows show the results for interaction between social context and time point for each of the attachment anxiety groups. The last two rows show the results for the interaction between attachment anxiety groups and time point for the social and non-social run of the task, separately.

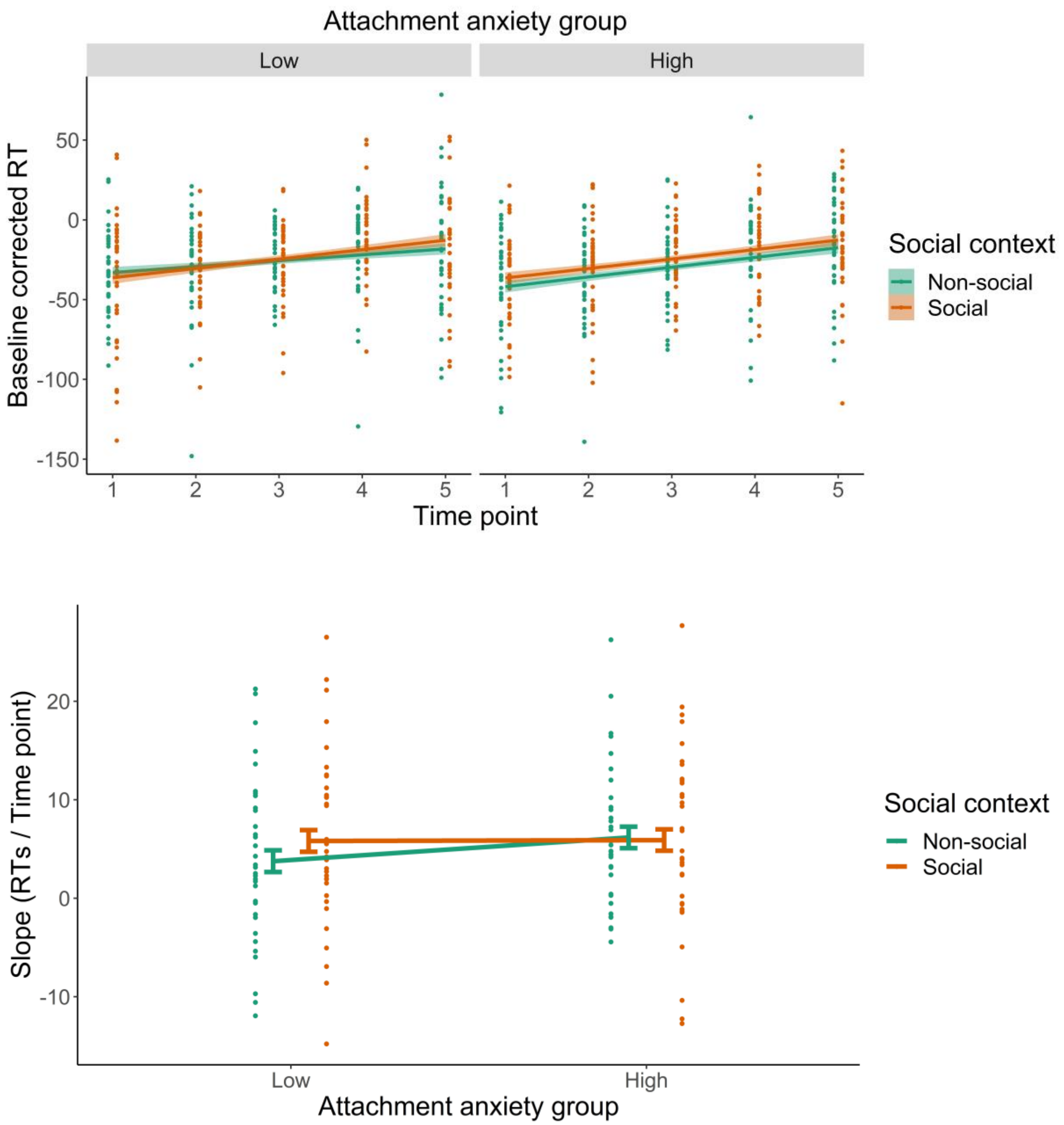

Figure S3. Effects of attachment anxiety and social context on PPS, related to Figure 3. Top panel: Baseline-corrected RTs across the time/distance points of tactile stimulation in the low (left) and high (right) attachment anxiety groups, as a function of social context. The shading surrounding each line represents the 95% confidence interval. Bottom panel: Slope of the baseline corrected RTs over the time/distance points of tactile stimulation, as a function of attachment anxiety groups (Low and High) and Social context (Social - orange and Non-social – green).

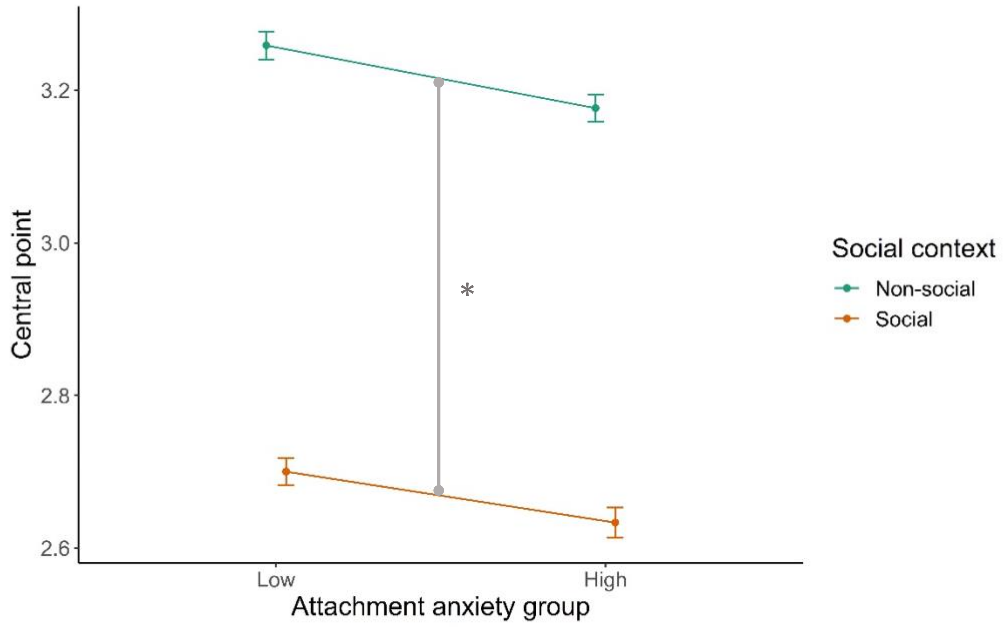

Figure S4: Sigmoid's central points in both attachment anxiety groups (Low and High) for each Social condition (Social and Non-social), related STAR methods. The two Groups in respect to attachment anxiety scores are displayed side by side, each containing two dots related to the social context (Social and Non-social). The dots correspondent to the social run are displayed in orange and are connected for visual facilitation between groups. The same can be said to the dots related to the Non-social run, displayed in green. The grey line and asterisks mean to highlight the significant difference in central point value between social contexts ($b_{(Social\ -\ Non\text{-}social)}$=-.55, SE=.21, p=.009).

Table S7. Full multilevel model results using the central point of the fitted sigmoid function as dependent variable in the step-wise model building, related to STAR Methods.

Effect	b	*SE*	*p*-value	Confidence intervals	
				Lower	Upper
Attachment anxiety group (High - Low)	-.05	.22	.809	-.48	.38
**Social context (Social - Non-social)**	**-.55**	**.21**	**.009**	**-.96**	**-.14**
Group X Social context	.02	.42	.967	-.81	.84

Table S8. Full multilevel model results using baseline corrected RTs as dependent variable in the step-wise model building, related to STAR Methods.

Effect	b	*SE*	*p*-value	Confidence intervals	
				Lower	Upper
STAI state	-.21	.22	.347	-.64	.22
**STAI state X Time point**	**.09**	**.05**	**.043**	**.00**	**.18**
STAI state X Time point X Group	-.18	.10	.077	-.38	.02
STAI trait	-.22	.20	.277	-.62	.18
STAI trait X Time point	.01	.04	.874	-.08	.09
STAI trait X Time point X Group	.08	.10	.463	-.13	.28
Trust scale	-3.81	4.03	.344	-11.71	4.08
Trust scale X Time point	-.59	.84	.482	-2.23	1.05
**Trust scale X Time point X Group**	**-5.50**	**1.72**	**.001**	**-8.87**	**-2.14**
Social status diff.	1.60	1.64	.331	-1.62	4.82
**Social status diff. X Time point**	**1.55**	**.34**	**<.001**	**.88**	**2.22**
Social status diff. X Time point X Group	-1.11	.74	.134	-2.56	.34

Note. Data was filtered to include only the social condition of the task (i.e. the only condition in which the confederate was present and therefore these variables are of relevance) and tested if the collected questions/questionnaires scores influenced PPS as a whole or influenced any of the previously-found interactions of interest; See Figure S5(A) for the follow-up of the STAI state x time point interaction; Figure S5(B) for the follow up of the social status x time point interaction; see Figure S5(C) and S5(D) for the follow up of the trust scale x time point x Group.

Table S9. Full multilevel model results using baseline corrected RTs as dependent variable in the step-wise model building, related to STAR Methods

Effect	b	*SE*	*p*-value	Confidence intervals	
				Lower	Upper
STAI state	<-.00	.01	.702	-.03	.02
STAI state X Group	.04	.03	.164	-.02	.09
STAI trait	<.00	.01	.983	-.02	.02
STAI trait X Group	**.06**	**.03**	**.041**	**.00**	**.11**
Trust scale	.22	.24	.345	-.24	.68
Trust scale X Group	-.54	.49	.271	-1.49	.42
Social status diff.	-.07	.09	.452	-.26	.11
Social status diff. X Group	-.25	.20	.215	-.65	.15

Note. As above, data was filtered to include only the social condition of the task (i.e. the only condition in which the confederate was present and therefore these variables are of relevance) and tested if the collected questions/questionnaires scores influenced PPS as a whole or influenced any of the previously-found interactions of interest.

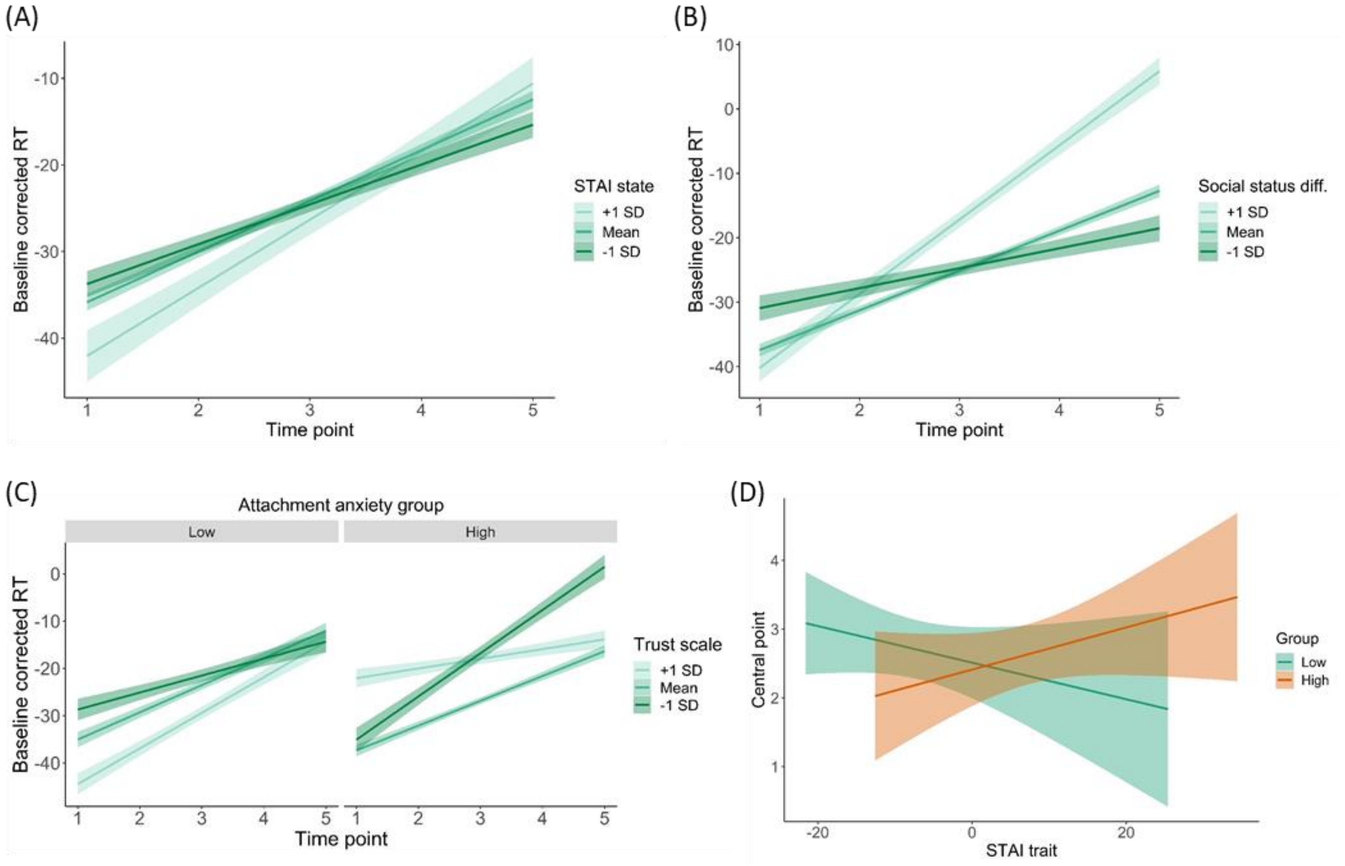

Figure S5: Statistically significant exploratory results of anxiety, perceived trustworthiness, and social status over social peripersonal space, related to STAR Methods. (A). Effect of STAI state on social peripersonal space (b=.09, SE=.05, p=.043), the higher the score on the STAI state questionnaire, greater the differentiation between close and far space. (B). Effect of the difference in perceived social status (participant-confederate) on social peripersonal space (b=1.55, SE=.34, p<.001), the more a participant perceived their social status to be higher than the confederate's social status, greater the differentiation between close and far space. (C). Effect of trust on social peripersonal space dependent on attachment anxiety group (b=-5.50, SE=1.72, p=.001), In the low attachment anxiety group, the higher the general trust scale score, greater the differentiation between close and far space. On the other hand, in the high attachment anxiety group, the higher the general trust scale score, smaller the differentiation between close and far space. (D). Effect of STAI trait on social PPS boundary dependent on attachment anxiety group (b=.06, SE=.03, p=.041), In the low attachment anxiety group, the higher the STAI trait score, closer to the body the PPS border is. On the other hand, in the high attachment anxiety group, the higher the STAI trait score, furthest away from the body the PPS border is. The shading surrounding each line represents the 95% confidence interval. The colour grading going from light to dark correspondent to high to low (+1 SD, Mean, -1 SD) scores on the correspondent questionnaire.

Table S10. Results of the full multilevel model testing the relation between attachment style and interpersonal space, controlling for developmental touch history and closeness, related to STAR Methods.

Predictors	*b*	SE	95% CI	Marginal $R^2_{(diff)}$	*p*-value
ANX	-.04	.01	[-.05, -.03]	.003	< .001***
AVO	.08	.01	[.07, .09]	.008	< .001***
DTH	-.03	.01	[-.05, -.02]	.002	< .001***
CLO	-.08	.01	[-.09, -.06]	.004	< .001***
AGE	-.01	< .00	[-.01, -.00]	.005	< .001***
ANX × AVO	< -.00	< .00	[-.01, .01]	< .000	.482
ANX × DTH	< .00	< .00	[-.01, .01]	< .000	.865
AVO × DTH	< .00	< .00	[-.00, .01]	< .000	.272
ANX × CLO	.01	.01	[.00, .03]	.001	.010*
AVO × CLO	-.02	.01	[-.03, -.01]	.001	.001**
Random effects					
Week ICC	.001				
Sexuality ICC	.004				
Ethnicity ICC	.006				
Observations	19353				
Marginal R^2 / Conditional R^2	.030 / .045				

Abbreviations: ANX, attachment anxiety; AVO, attachment avoidance; DTH, developmental touch history; CLO, closeness. Significance: * < .05; ** < .01; *** < .001.

Table S11. Results of the full multilevel model testing the relation between interoceptive accuracy and interpersonal space, controlling for attachment style, related to STAR Methods.

Predictors	*b*	SE	95% CI	Marginal $R^2_{(diff)}$	*p*-value
IAC	-.01	.01	[-.04, .02]	< .000	.468
ANX	-.03	.01	[-.04, -.02]	.002	< .001***
AVO	.11	.01	[.10, .12]	.017	< .001***
AGE	< -.00	< .00	[-.01, -.00]	.004	< .001***
ANX × AVO	< -.00	< .00	[-.01, .00]	< .000	.230
ANX × IAC	.01	.01	[-.01, .03]	< .000	.479
AVO × IAC	-.02	.01	[-.04, .00]	< .000	.090
Random effects					
Week ICC			.001		
Sexuality ICC			.004		
Ethnicity ICC			.007		
Gender ICC			.001		
Observations			19227		
Marginal R^2 / Conditional R^2			.022 / .036		

Abbreviations: ANX, attachment anxiety; AVO, attachment avoidance; IAC, interoceptive accuracy. Significance: * < .05; ** < .01; *** < .001.

Table S12. Results of the full multilevel model testing the relation between attachment style and interpersonal space, controlling for developmental touch history and closeness, related to STAR Methods.

Predictors	*b*	SE	95% CI	Marginal $R^2_{(diff)}$	*p*-value
CON	.03	.01	[.02, .05]	.001	< .001***
EXT	-.15	.01	[-.16, -.14]	.029	< .001***
OPE	-.01	.01	[-.02, .01]	< .000	.412
NEU	.02	.01	[.01, .03]	< .000	.001**
AGR	-.05	.01	[-.06, -.03]	.001	< .001***
ANX	-.05	.01	[-.06, -.04]	.003	< .001***
AVO	.06	.01	[.05, .07]	.004	< .001***
AGE	< -.00	< .00	[-.00, -.00]	.002	< .001***
ANX × AVO	< -.00	< .00	[-.01, .01]	< .000	.601
ANX × CON	< -.00	.01	[-.01, .01]	< .000	.502
AVO × CON	< .00	.01	[-.01, .01]	< .000	.601
ANX × EXT	.01	< .00	[-.00, .01]	< .000	.215
AVO × EXT	< -.00	< .00	[-.01, .01]	< .000	.621
ANX × OPE	< .00	.01	[-.01, .01]	< .000	.395
AVO × OPE	< .00	.01	[-.01, .02]	< .000	.445
ANX × NEU	< .00	< .00	[-.00, .01]	< .000	.354
AVO × NEU	< -.00	< .00	[-.01, .01]	< .000	.873
ANX × AGR	.01	.01	[-.00, .02]	< .000	.170
AVO × AGR	.01	.01	[-.00, .02]	< .000	.176
Random effects					
Week ICC			.001		
Sexuality ICC			.004		
Ethnicity ICC			.006		
Observations			19356		
Marginal R^2 / Conditional R^2			.063 / .076		

Abbreviations: ANX, attachment anxiety; AVO, attachment avoidance; CON, conscientiousness; EXT, extroversion; OPE, openness; NEU, neuroticism; AGR, agreeableness. Significance: * < .05; ** < .01; *** < .001.

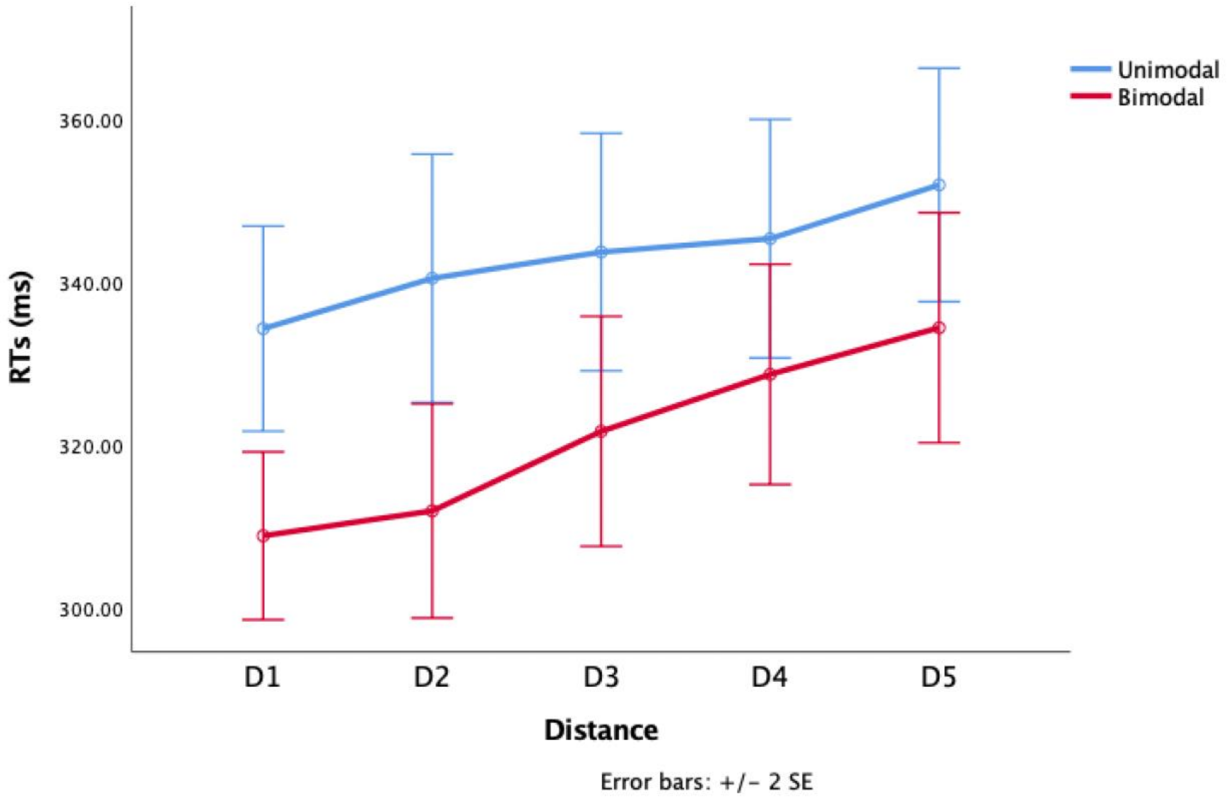

Figure S6. RTs of bimodal and unimodal trial across the five distances in Study 1, related to STAR Methods. Averaging across touch and PPS group condition, the mean RTs to uni-modal and bi-modal trials for each time interval of the tactile stimulation (i.e., "distance") were calculated and entered in a repeated measure ANOVA with Modality (uni-modal tactile *vs* bi-modal visuo-tactile trials) and Distance of the virtual object (D1, D2, D3, D4, D5, D6) as within-subject factors. The main effect of Modality [$F(1, 47) = 112.58$, $p < .001$, $\eta_p^2 = .71$], and distance [$F(4, 188) = 40.57$, $p < .001$, $\eta_p^2 = .46$] were significant, and as expected, qualified by a two-way Modality X Distances interaction [$F(4, 188) = 5.75$, $p = .001$, $\eta_p^2 = .12$]. To better understand this interaction we calculated the difference score between unimodal and bimodal trials (bimodal – unimodal) and examined whether there were differences between distances. As expected, planned comparisons suggest no difference between the closer distances to the body (i.e., D1 vs D2, D1 vs D3, p's > .265), but a significant difference between closer distances to the body and those farther away (i.e., D1 vs D4, D1 vs D5, p < .003), indicating a larger difference between unimodal and bimodal trials at closer, relative to farther, distances from the body. Given that one would expect faster RTs in response to visuo-tactile (bimodal) stimuli relative to tactile (unimodal) on distances closer to the body, i.e., multisensory boosting effect, these results indicate that our PPS task was successful.

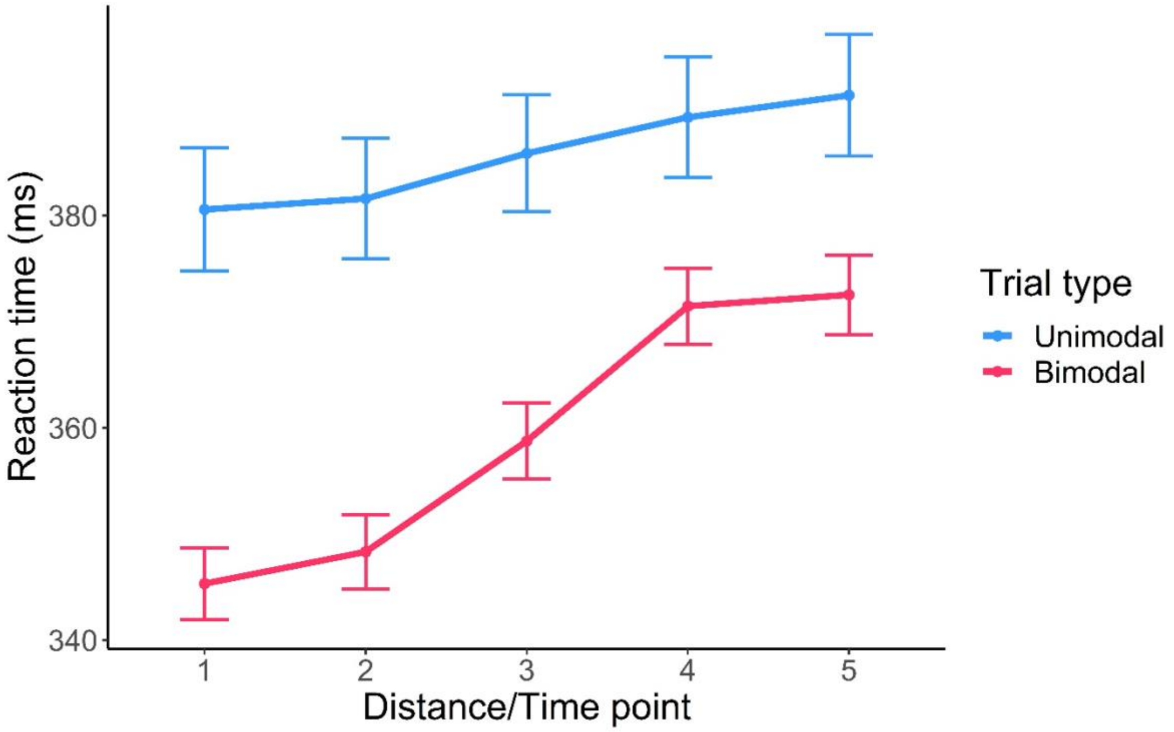

Figure S7. RTs of bimodal and unimodal trial across the five distances in Study 2, related to STAR Methods, error bars +/- 2 SE. As in study 1, we verified that Modality is a statistically significant factor when predicting reaction times, as expected. We tested the effect of modality, time point of stimulation and their interaction in a repeated measures ANOVA (both factors entered as within subject). The main effect of Modality [$F(1, 19225) = 335.82$, $p < .001$, $\eta p2 = .017$], and distance/time point [$F(4, 19225) = 43.88$, $p < .001$, $\eta p2 = .009$] were significant, and was well as their interaction [$F(4, 19225) = 6.182$, $p < .001$, $\eta p2 = .001$].

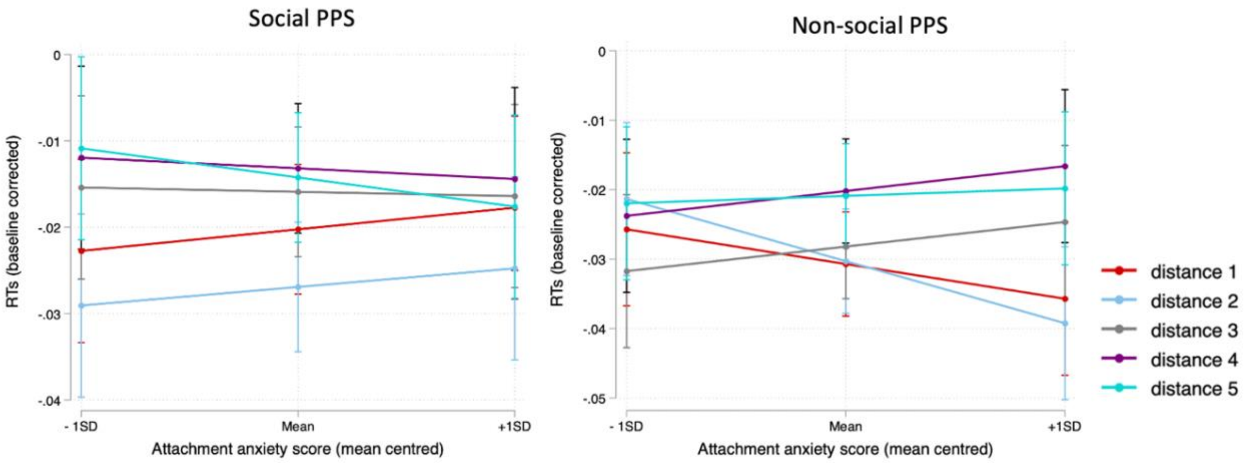

Figure S8. The effect of attachment anxiety, distance and PPS group on RTs (i.e., data before fitting), related to STAR Methods. We conducted a mixed model on the RTs (baseline corrected) specifying PPS group (social, non-social) and distance (D1, D2, D3, D4, D5) as dummy-coded categorical predictors and attachment anxiety, we specified attachment anxiety as a continuous predictor. We included all interaction terms, while controlling for attachment avoidance. As in the main text (analyses conducted on the slopes instead of RTs) we observed the equivalent interaction of distance x PPS group x attachment anxiety also on RTs. Indeed, the opposite direction RTs modulation for closer and farther distances between the social and non-social PPS as a function of attachment anxiety follows the same pattern as the slopes described in the main text.
